# Is YouTube™ a Reliable Source of Information for the Current Use of HIPEC in the Treatment of Ovarian Cancer?

**DOI:** 10.3390/cancers17193222

**Published:** 2025-10-02

**Authors:** Francesco Mezzapesa, Elisabetta Pia Bilancia, Margarita Afonina, Stella Di Costanzo, Elena Masina, Pierandrea De Iaco, Anna Myriam Perrone

**Affiliations:** 1 Department of Medical and Surgical Sciences (DIMEC), University of Bologna, 40138 Bologna, Italystella.dicostanzo@aosp.bo.it (S.D.C.); elena.masina3@studio.unibo.it (E.M.); pierandrea.deiaco@unibo.it (P.D.I.); myriam.perrone@unibo.it (A.M.P.); 2 Division of Oncologic Gynecology, IRCCS Azienda Ospedaliero—Universitaria di Bologna, 40138 Bologna, Italy; 3 Department of Obstetrics and Gynecology, San Paolo Hospital Medical School, University of Milan, 20142 Milan, Italy; afoninamargarita@hotmail.com

**Keywords:** YouTube™, ovarian cancer, hyperthermic, HIPEC, PEMAT, DISCERN

## Abstract

**Simple Summary:**

YouTube™ is one of the most widely used platforms for health-related information, yet the quality of its medical content remains unexamined. In this study, we systematically reviewed YouTube™ videos discussing Hyperthermic Intraperitoneal Chemotherapy (HIPEC) in the management of advanced ovarian cancer to assess their reliability and educational value for both patients and healthcare professionals. Our findings show that while professional-targeted videos provide more comprehensive and accurate information, patient-focused videos are often less reliable and omit critical details such as treatment indications, risks, and clinical outcomes. These results emphasize the importance of improving the quality of online cancer education and highlight the need for accurate digital resources to better support patient decision-making and professional learning.

**Abstract:**

**Introduction**: YouTube™ is a widely accessible platform with unfiltered medical information. This study aimed to evaluate the educational value and reliability of YouTube™ videos on Hyperthermic Intraperitoneal Chemotherapy (HIPEC) for advanced epithelial ovarian cancer treatment. **Methods**: YouTube™ videos were searched using the keywords “*ovarian cancer*”, “*debulking surgery*”, “*hyperthermic*”, and “*HIPEC*”. Patient Education Materials Assessment Tool for Audiovisual Content (PEMAT A/V) score, DISCERN, Misinformation Scale, and the Global Quality Scale (GQS) were employed to assess the clarity, quality, and reliability of the information presented. **Results**: Of the 150 YouTube™ videos screened, 71 were suitable for analysis and categorized by target audience (general public vs. healthcare workers). Most (57, 80.2%) were uploaded after the “Ov-HIPEC” trial (18 January 2018), with a trend toward more videos for healthcare workers (*p* = 0.07). Videos for the general public were shorter (*p* < 0.001) but received more views (*p* = 0.06) and likes (*p* = 0.09), though they were of lower quality. The DISCERN score averaged 50 (IQR: 35–60), with public-targeted videos being less informative (*p* < 0.001), a trend mirrored by the Misinformation Scale (*p* < 0.001) and GQS (*p* < 0.001). The PEMAT A/V scores showed 80% Understandability (IQR: 62–90) and 33% Actionability (IQR: 25–100), with no significant difference between groups (*p* = 0.15, *p* = 0.4). **Conclusions**: While YouTube™ provides useful information for healthcare professionals, it cannot be considered a reliable source for patients seeking information on HIPEC for ovarian cancer. Many videos contribute to misinformation by not properly explaining treatment indications, timing, adverse effects, multimodal approaches, or clinical trial findings.

## 1. Introduction

Epithelial ovarian cancer (EOC) is the most lethal of all gynecological malignancies, with approximately 80% of cases diagnosed at advanced stages [1]. This is largely due to the unspecific abdominal symptoms, which often lead to delays in diagnosis [2]. The current standard of care consists of complete cytoreduction surgery followed by a platinum-based chemotherapy regimen and maintenance therapy [3]. In cases when complete cytoreduction is not initially feasible, neoadjuvant chemotherapy (NACT) is recommended to reduce tumor burden and facilitate optimal surgical resection [4,5].

EOC primarily spreads within the peritoneal cavity and rarely disseminates via the bloodstream [6]. This feature has led to the development of innovative strategies for delivering chemotherapy directly into the peritoneal cavity [7]. Among these, hyperthermic intraperitoneal chemotherapy (HIPEC) has garnered increasing attention after the publication in 2018 by Van Driel et al. of a multicenter prospective trial (OV-HIPEC-1), demonstrating that the addition of HIPEC to interval debulking surgery resulted in an improvement of 4 months in progression-free survival (PFS) and 12 months overall survival (OS) [8]. This finding sparked renewed interest in the potential role of HIPEC in different settings of treatment [9]. The Italian prospective HORSE trial and the phase II trial by the Memorial Sloan Kettering Team Ovary did not show a significant impact of HIPEC on PFS in patients undergoing secondary debulking surgery for platinum-sensitive peritoneal recurrence [10,11]. In contrast, the French CHIPOR trial demonstrated a survival benefit when HIPEC was administered as a consolidation therapy following six cycles of platinum-based chemotherapy and cytoreductive surgery in patients with platinum-sensitive recurrent EOC [12]. Finally, a meta-analysis by Della Corte et al. [13], including 3 retrospective studies, found no advantage in PFS adding HIPEC to patients undergoing primary debulking surgery. However, the results of the ongoing OV-HIPEC-2 trial (NCT03772028), expected in 2026, will be crucial in further clarifying its role in the upfront setting [14].

While the scientific community continues to debate the optimal setting for integrating HIPEC into EOC treatment, patients facing an advanced-stage disease diagnosis often seek out any possibility that may reduce their symptoms and ultimately offer hope. With the increasing accessibility of health-related content online, social media has become a widely used resource for medical information. YouTube™, which has over 2.5 billion monthly users and more than 5 billion videos viewed daily, represents a significant, free and easily accessible platform for disseminating health-related content [15]. However, standardized methods for assessing the quality and reliability of medical information are lacking, resulting in a mix of accurate, incomplete, and potentially misleading content [16]. This study aims to evaluate the quality of information, defined in terms of reliability and educational value, of YouTube™ videos on the use of HIPEC in advanced EOC.

## 2. Materials and Methods

### 2.1. Video Search and Inclusion Criteria

In accordance with PRISMA guidelines, we conducted a systematic search on YouTube™ on 31 August 2024, at 10:00 a.m. (CEST). The search was performed on personal computers; however, to minimize potential biases, a private browsing session (incognito mode) was used while logged out of any personal accounts. For the search filter, we used the following keywords: ‘*ovarian cancer*,’ ‘*debulking surgery*,’ ‘*hyperthermic*,’ and ‘*HIPEC*.’ These terms were chosen by the authors with the intention of mirroring the vocabulary that patients may use, while deliberately avoiding more technical terminology that could bias the results toward highly specialized content.

Videos were included regardless of their publication date on the YouTube platform, based on the following criteria: (I) content presented in English and (II) discussion of HIPEC in the context of ovarian cancer. According to these criteria, videos were independently screened, selected and assessed by two authors (FM, EB), and any discrepancies were resolved by a senior author (AMP) [17,18].

For each eligible video, we recorded the following variables: video duration (in seconds), number of views, persistence time on YouTube™ (in days), gender of the narrating voice, Continent of origin, likes, comments, channel subscribers, view ratio (calculated as the number of views divided by the persistence time), the number of videos with disabled comments, like/view ratio (calculated as the number of likes divided by the number of views per video) and comment/view ratio (calculated as the number of comments divided by the number of views per video).

Furthermore, we collected additional information on the author (private individual vs. institution), date of upload, and target audience (general public vs. healthcare professionals). When the target audience was not explicitly stated, allocation was determined based on the language register used, the type and level of content detail, and the profile of the uploader.

### 2.2. Strategies and Tools for Video Content Assessment

All included videos were blindly evaluated by two authors, both senior residents with a research focus in gynecologic oncology, in terms of educational value and reliability of information using four freely available tools, in line with previous studies [19,20]. To ensure consistency in scoring, the two authors first independently reviewed the user guides provided online for PEMAT A/V (https://www.ahrq.gov/health-literacy/patient-education/pemat2.html, accessed on 31 August 2024) and DISCERN (https://www.ndph.ox.ac.uk/research/research-groups/applied-health-research-unit-ahru/discern, accessed on 31 August 2024) and subsequently discussed and calibrated their interpretations.

The Patient Education Materials Assessment Tool for Audiovisual Content (PEMAT A/V) score was used to evaluate and compare the understandability and actionability of educational content [21]. This tool consists of 17 items, with 13 assessing understandability (items 1–13) and four evaluating actionability (items 14–17). Each item was rated using three response options: *agree* (1 point), *disagree* (0 points), or *not available* (NA) [22]. All items were applied consistently across videos, as YouTube videos are universally accessible and can therefore serve as a potential source of patient education, regardless of the target audience.

The total score was determined through a three-step process. After summing the points assigned to Understandability or Actionability, each total was divided by the number of rated items, excluding any marked as NA. Finally, the result was multiplied by 100. The final scores were presented as percentages, with higher values reflecting greater understandability and/or actionability of the content.

DISCERN is a standardized questionnaire used to evaluate the quality of information on treatment options for health conditions [23]. It comprises 16 items that assess the material’s reliability (Section 1, 8 items), the quality of information on treatment choice (Section 2, 7 items) and the overall rating of the source (Section 3, 1 item). Each item is rated on a 5-point Likert scale, ranging from 1 (*Poor quality*) to 5 (*High quality*). Based on the total score, materials are categorized as *excellent* (63–80), *good* (51–62), *fair* (39–50), *poor* (27–38), or *very poor* (16–26) [24].

The Global Quality Score (GQS) is a tool for assessing the quality, feasibility, and utility of a video. Five possible scores from 1 (poor quality) to 5 (excellent quality) were assigned.

A specific misinformation tool was designed to assess discrepancies between video content and current scientific knowledge on this topic, following a previously established methodology [20]. In particular the questions were developed based on the European Society of Gynecologic Oncology (ESGO) recommendations on the use of HIPEC [3]. Scores range from 1 (extreme misinformation) to 5 (no misinformation), with lower scores indicating a greater deviation from established evidence. The following questions were developed: *Q1 “In which clinical scenarios should HIPEC be considered?; Q2 “What are the potential complications associated with the HIPEC procedure?”; Q3 “Should HIPEC be performed in high-volume centers with specialized expertise?”; Q4 “Is the impact of HIPEC on survival outcomes clarified?”; Q5 “Should HIPEC be offered only within the context of a randomized controlled trial (RCT)?”*

### 2.3. Statistical Analyses

Descriptive statistics were presented as medians and interquartile ranges (IQR) for continuous variables or counts and percentages for categorical variables. Comparisons between groups were performed using Welch Two Sample *t*-test for normally distributed continuous variables, Wilcoxon rank-sum test for non-normally distributed continuous variables, Pearson’s chi-squared test and Fisher’s exact test as appropriate. Inter-rater reliability (IRR) between the two authors’ evaluations was assessed using Cohen’s kappa coefficient; if IRR was below 0.8, a third evaluation by a senior author was planned to be performed. Given the descriptive nature of the study, no formal sample size or power calculation was performed. All analyses were conducted using the R software environment (R version 4.0.0), and all tests were two-sided with significance set at *p* < 0.05.

## 3. Results

**Figure 1:**
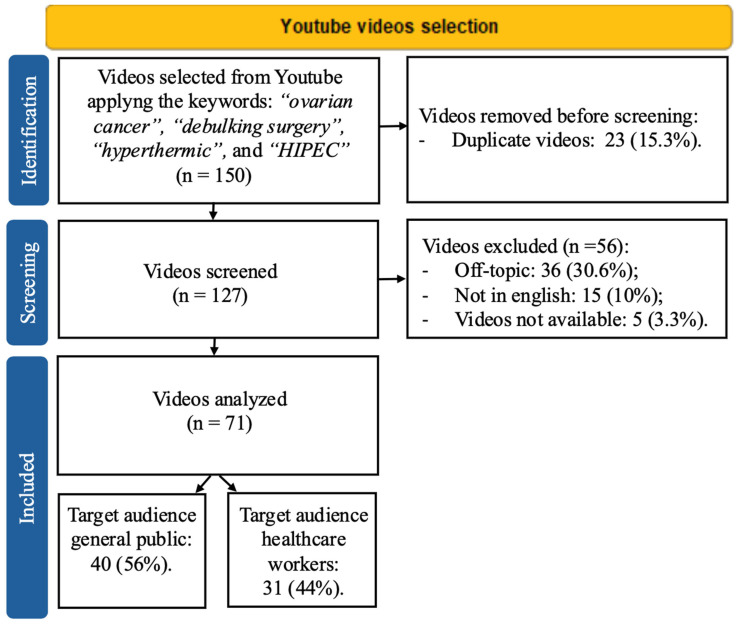
Flow chart of Video selection.

A total of 150 videos were collected and screened through the inclusion criteria. After the exclusion of duplicate videos (n = 23, 15.3%), 127 videos were assessed for eligibility. The following exclusion criteria were applied: off-topic videos (n = 36, 30.6%), non-English language videos (n = 15, 10%), and video not available (n = 5, 3.3%). A total of 71 (47.3%) videos were eligible for the analyses (Figure 1).

The videos included were categorized based on their target audience: 40 (56%) were aimed at the general public, while 31 (44%) were intended for healthcare workers. A significant difference was observed in video length, with a longer median duration for videos targeting healthcare workers (710 vs. 174 s, *p* < 0.001). No other statistically significant differences were found in the videographic characteristics (Table 1).

Among the 71 videos, 10 (14%) were published by members of the general public. Of These, 7 (18%) were directed at the general audience and 3 (9.7%) at the healthcare professionals. Meanwhile, 61 (86%) of the videos were published by healthcare professionals, with 33 (54.1%) targeting the general public and 28 (45.9%) aimed at healthcare workers.

The median PEMAT A/V Understandability score was 80% (IQR: 62–90) with a IRR of 0.87, and without a significant difference between videos directed at the general public and those targeting healthcare workers (80% vs. 77%, *p* = 0.15). Similarly, the median PEMAT A/V Actionability score was 33% (IQR: 25–100), with no significant difference based on the target audience (50% vs. 33%, *p* = 0.4) (Table 2).

According to the DISCERN score, the median score for Section 1 was 27 (IQR: 20–33), with videos targeting healthcare workers scoring significantly higher than those aimed at the general public (32 vs. 21, *p* < 0.001). The median score for Section 2 was 19 (IQR: 16–24), again higher for healthcare worker-directed content (21 vs. 17, *p* = 0.001). The total DISCERN score had a median of 50 (IQR: 35–60), with videos for healthcare professionals scoring significantly higher than those for the general public (56 vs. 40, *p* < 0.001). The IRR was 0.92.

Based on the GSQ (Global Score Quality) rating, 7% (n = 5) of the videos were classified as excellent, 35% (n = 25) as good, 30% (n = 21) as moderate, 18% (n = 13) as generally poor, and 9.9% (n = 7) as poor. When stratified by target audience, 16% of videos for healthcare workers were rated excellent compared to 0% for the general public. Additionally, 55% vs. 20% were rated as good, 26% vs. 33% as moderate, 3.2% vs. 30% as generally poor, and 0% vs. 18% as poor quality. The overall IRR was 0.85.

Regarding the misinformation score, the IRR was 0.97. Among the 71 selected videos, 50 (70.4%) addressed Misinformation Question 1, with 19 (38%) targeting the general public and 31 (62%) targeting healthcare professionals. Misinformation Question 2 was addressed by 23 videos (32.4%), including 5 (21.7%) intended for the general public and 18 (78.3%) for healthcare professionals. Misinformation Question 3 was covered in 28 videos (39.4%), with 8 (28.6%) directed at the general public and 20 (40%) at healthcare professionals. Misinformation Question 4 was addressed in 41 videos (57.7%), of which 16 (39%) were aimed at the general public and 25 (61%) at healthcare professionals. Finally, Misinformation Question 5 was covered in 17 videos (23.9%), with only 1 (5.9%) targeting the general public and 16 (94.1%) targeting healthcare professionals. Overall, videos intended for healthcare professionals were significantly more likely to address misinformation-related topics than those aimed at the general public.

A further subanalysis was conducted based on the gender of the narrating voice used in the videos (Female, Male, Both or No voice). Results are reported in the Supplementary Materials (Table S1). Videos recorded with a female voice had lower DISCERN scores compared to those with a male voice or both voices (46 vs. 51 vs. 58, respectively), but higher scores than videos with no voice (*p* = 0.020). Similarly, the GQS was higher in the male and both voices groups compared to the female group (*p* = 0.013). No differences were observed in PEMAT A/V Understandability (*p* = 0.918), Actionability (*p* = 1.0), or Misinformation scores (*p* = 0.106). We believe, however, that these differences may be influenced by the higher proportion of videos targeting the general population in the female voice group compared with the male voice group (68% vs. 54%, *p* = 0.099).

## 4. Discussion

**Figure 2:**
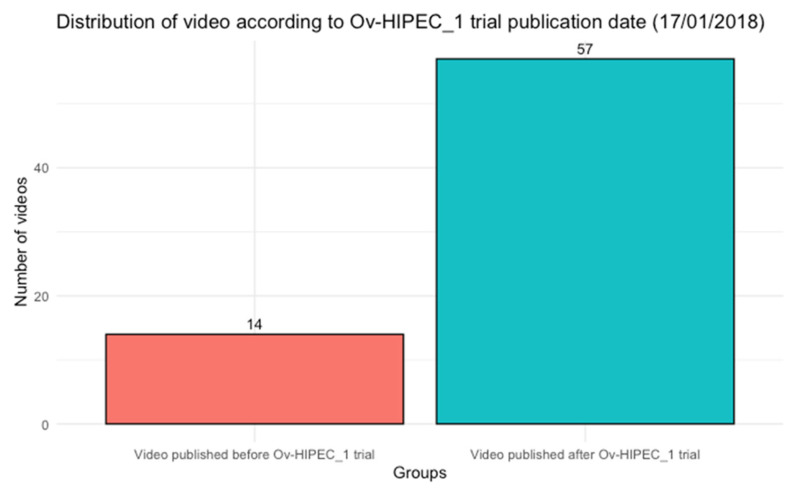
Distribution of the number of video published on YouTube according to the publishing date of “Cytoreductive surgery with or without hyperthermic intraperitoneal chemotherapy in patients with advanced ovarian cancer (OVHIPEC-1)” (17 January 2018).

To the best of our knowledge, this is the first study to systematically evaluate the educational value and information reliability of YouTube™ videos on the use of HIPEC in the treatment of EOC. Our findings indicate that videos targeting healthcare professionals generally achieved higher scores and contained less misinformation compared to those aimed at the general public. Interestingly, most videos (n = 57, 80.3%) on this topic were uploaded after the publication of the OV-HIPEC-1 trial [8] (Figure 2). Since its publication, the number of studies investigating HIPEC in different settings of ovarian cancer treatment has increased, yielding inconsistent results [9,12,25]. The uncertainty surrounding HIPEC is also reflected in international guidelines. While organizations such as the National Comprehensive Cancer Network (NCCN), the American Society of Clinical Oncology (ASCO), the British Gynecological Cancer Society (BGCS), the China Anti-Cancer Association (CACA) and the Taiwanese society acknowledge its use in the IDS setting and recommend its application in highly specialized centers [4,26,27], the European Society of Gynecologic Oncology and the European Society of Medical Oncology (ESGO/ESMO) do not recognize HIPEC as a standard treatment and advise its use only within randomized clinical trials (RCTs) [3]. The same recommendation is given by the Japan Society of Gynecology Oncology (JSGO) [28].

Notably, no specific guidelines exist for Southeast Asian, South American, or African countries, likely due to the high complexity and costs associated with this technology [29].

Most videos were uploaded by healthcare professionals but intended for a general audience. However, our analysis suggests that the overall reliability and educational value of these videos is limited. This is particularly concerning, as healthcare professionals are typically able to critically appraise the accuracy of the information presented, whereas patients and caregivers may struggle to distinguish between reliable content and misleading or incomplete information, especially when produced by non–healthcare professionals [30,31,32].

Using the PEMAT A/V tool, we found no significant difference in Understandability and Actionability scores between videos targeting different audiences. The Understandability score reflects how well viewers can process and explain the key messages presented, while the Actionability score assesses whether viewers can apply the information provided. Scores below 70% are considered poorly understandable or actionable [21]. In our study, the median Understandability score was higher (80% for the general public and 77% for healthcare professionals, *p* = 0.15), suggesting that the messages conveyed were generally comprehensible. In line with previous literature [33,34] the median Actionability score was significantly lower (33% for the general public and 50% for healthcare professionals, *p* = 0.4), indicating that while the information was understandable, its practical application remained unclear. Similarly, all three sections of the DISCERN tool were significantly higher in videos targeting healthcare professionals. This suggests that content designed for a professional audience was generally more detailed and of higher educational value. Comparable results were reported in a recent study by Ting Xu et al. on vulvar cancer videos on YouTube™ [35]. These findings were also consistent with the assessments obtained using the GQS and misinformation evaluation tools, further reinforcing the discrepancy in content reliability based on the target audience. However, despite higher scores in videos targeting healthcare professionals, concerns remain regarding their overall educational value. Previous studies have indicated that even high-quality YouTube™ content may be insufficient for the education of residents or fellows [36,37], leading some authors to suggest that videos should undergo professional evaluation prior to publication [38]. However, given the vast scope of medical topics and the unrestricted nature of video uploads on social media platforms, implementing comprehensive content regulation remains challenging. Instead, a more feasible approach may involve developing social policies aimed at equipping patients and their relatives with the necessary tools to critically assess the reliability of online health information [39]. Additionally, the medical community should acknowledge the increasing reliance of oncological patients on online resources [40].

This study has some limitations. First, YouTube™ search results are influenced by the platform’s search algorithms, which tailor results based on users’ previous search activities. To mitigate this bias, all searches were conducted in incognito mode. Moreover, videos were evaluated independently of their source, reducing potential allocation bias. Second, the deliberate choice of search keywords may have introduced selection bias, potentially excluding other relevant reliable or unreliable content. Third, as YouTube™ is a continuously evolving platform, our sample reflects only the quality of videos available at the specific date and time of data collection. Lastly, the use of DISCERN for audiovisual material has not been formally validated, although it is widely applied in the literature. In addition, no officially validated thresholds have been established for either PEMAT A/V or DISCERN; however, the thresholds adopted in this study are consistent with those reported in previous studies [18]. Despite these limitations, our study is the first to systematically evaluate the quality of YouTube™ videos on the use of HIPEC in ovarian cancer, providing valuable insights into the type of information accessible to patients and healthcare professionals. Future steps may aim to generate high-quality videos with improved and reliable information for patients, produced by medical centers and alliance groups. These efforts could guide patients toward high-quality information on social media and provide them with comprehensive resources to support truly informed consent. Lastly, future studies may aim to investigate another niche and not widely adopted procedure such as Pressurized Intraperitoneal Aerosol Chemotherapy (PIPAC) [41].

## 5. Conclusions

In conclusion, the overall educational value and reliability of YouTube™ videos on HIPEC in ovarian cancer treatment, within the sample analyzed in this study, is low, with only slightly better content available for healthcare professionals. Our findings highlight the need for more comprehensive and reliable video content on this topic to ensure that patients seeking information on YouTube™ receive accurate and well-balanced information.

## Figures and Tables

**Table 1:** Video graphic characteristics.

Characteristic	Overall, n = 71 ^1^	TARGET.AUDIENCE General Public, n = 40 (56%) ^1^	TARGET.AUDIENCE Healthcare Workers, n = 31 (44%) ^1^	*p*-Value ^2^
Length of the video (seconds)	285 (141, 695)	174 (113, 314)	710 (304, 2533)	<0.001
Views (n)	608 (244, 2085)	743 (345, 3684)	425 (213, 1064)	0.069
Author Sex				0.107
Male	39 (55%)	18 (58%)	21 (53%)	
Female	19 (27%)	6 (19%)	13 (33%)	
Both	10 (14%)	7 (23%)	3 (7.5%)	
No voice	3 (4.2%)	0 (0%)	3 (7.5%)	
Continent of Origin				0.233
North America	31 (48%)	13 (42%)	18 (53%)	
Asia	23 (35%)	9 (29%)	14 (41%)	
Europe	4 (6.2%)	3 (9.7%)	1 (2.9%)	
Africa	1 (1.5%)	1 (3.2%)	0 (0%)	
Mixed origin	6 (9.2%)	5 (16.2%)	1 (2.9%)	
Unknown	6	0	6	
Duration Day on YouTube (days)	1269 (809, 2260)	1497 (669, 2465)	1257 (1173, 2068)	>0.9
Likes (n)	6 (2, 16)	8 (3, 24)	4 (2, 10)	0.094
Likes/views ratio	0.008 (0.004, 0.012)	0.009 (0.006, 0.015)	0.006 (0.004, 0.010)	0.202
Subscribers (n)	10,500 (1361, 29,350)	10,500 (801, 58,975)	10,800 (1920, 22,600)	0.7
Comments (n)				0.8
abled	58 (82)	33 (83)	25 (81)	
disabled (as per YouTube policy)	13 (18)	7 (18)	6 (19)	
Comments/views ratio	0 (0.0000, 0.0010)	0 (0.0000, 0.0005)	0 (0.0000, 0.0013)	0.320
Unknown	13	7	6	
Author group (n)				0.5
General public	10 (14)	7 (18)	3 (9.7)	
Healthcare workers	61 (86)	33 (83)	28 (90)	

^1^ Median (IQR); n (%). ^2^ Fisher’s exact test; Wilcoxon rank sum test; Welch Two Sample *t*-test; Pearson’s Chi-squared test.

**Table 2:** Scoring tools results.

Characteristic	Overall, n = 71 ^1^	TARGET.AUDIENCE General Public, n = 40 (56%) ^1^	TARGET.AUDIENCE Healthcare Workers, n = 31 (44%) ^1^	*p*-Value ^2^
PEMAT_UNDERSTANDABILITY (%)	80 (62, 90)	80 (63, 94)	77 (62, 88)	0.15
PEMAT_ACTIONABILITY (%)	33 (25, 100)	33 (19, 100)	50 (33, 88)	0.4
DISCERN_Section 1 (n)	27 (20, 33)	21 (17, 27)	32 (28, 35)	<0.001
DISCERN_Section 2 (n)	19 (16, 24)	17 (11, 22)	21 (17, 26)	0.001
DISCERN_total score (n)	50 (35, 60)	40 (32, 52)	56 (51, 64)	<0.001
MISINFORMATION_Score (n)	2.00 (1.00, 3.50)	1.00 (0.00, 2.00)	3.00 (3.00, 5.00)	<0.001
Q1. In which clinical scenarios should HIPEC be considered?	50 (71.43)	19 (38)	31 (62)	
Q2. What are the potential complications associated with the HIPEC procedure?	23 (32.39)	5 (21.74)	18 (78.26)	
Q3 Should HIPEC be performed in high-volume centers with specialized expertise?	28 (39.44)	8 (28.57)	20 (40)	
Q4. Is the impact of HIPEC on survival outcomes clarified?	41 (57.75)	16 (9.02)	25 (60.98)	
Q5. Should HIPEC be offered only within the context of a randomized controlled trial (RCT)?	17 (23.94)	1 (5.88)	15 (88.24)	
GLOBAL_QUALITY_SCORE				<0.001
1	7 (9.9)	7 (18)	0 (0)	
2	13 (18)	12 (30)	1 (3.2)	
3	21 (30)	13 (33)	8 (26)	
4	25 (35)	8 (20)	17 (55)	
5	5 (7.0)	0 (0)	5 (16)	

^1^ Median (IQR); n (%); ^2^ Fisher’s exact test; Wilcoxon rank sum test; Welch Two Sample *t*-test; Pearson’s Chi-squared test.

## Data Availability

The data presented in this study are available on request from the corresponding author.

## Supplementary Materials

The following supporting information can be downloaded at: https://www.mdpi.com/article/10.3390/cancers17193222/s1, Table S1: Subanalysis according to Gender narrating voice of the video.

## References

[1] Ferlay J., Colombet M., Soerjomataram I., Parkin D.M., Piñeros M., Znaor A., Bray F. (2021). Cancer statistics for the year 2020: An overview. Int. J. Cancer.

[2] Chase D.M., Neighbors J., Perhanidis J., Monk B.J. (2021). Gastrointestinal symptoms and diagnosis preceding ovarian cancer diagnosis: Effects on treatment allocation and potential diagnostic delay. Gynecol. Oncol..

[3] Ledermann J., Matias-Guiu X., Amant F., Concin N., Davidson B., Fotopoulou C., González-Martin A., Gourley C., Leary A., Lorusso D. (2024). ESGO–ESMO–ESP consensus conference recommendations on ovarian cancer: Pathology and molecular biology and early, advanced and recurrent disease. Ann. Oncol..

[4] Gaillard S., Lacchetti C., Armstrong D.K., Cliby W.A., Edelson M.I., Garcia A.A., Ghebre R.G., Gressel G.M., Lesnock J.L., Meyer L.A. (2025). Neoadjuvant Chemotherapy for Newly Diagnosed, Advanced Ovarian Cancer: ASCO Guideline Update. J. Clin. Oncol..

[5] Perrone A.M., Coada C.A., Ravegnini G., De Leo A., Damiano G., De Crescenzo E., Tesei M., Di Costanzo S., Genovesi L., Rubino D. (2023). Post-operative residual disease and number of cycles of neoadjuvant chemotherapy in advanced epithelial ovarian carcinoma. Int. J. Gynecol. Cancer.

[6] Farsinejad S., Cattabiani T., Muranen T., Iwanicki M. (2019). Ovarian Cancer Dissemination—A Cell Biologist’s Perspective. Cancers.

[7] Kumar K.P., Madhusoodanan M., Pangath M., Menon D. (2025). Innovative landscapes in intraperitoneal therapy of ovarian cancer. Drug Deliv. Transl. Res..

[8] Van Driel W.J., Koole S.N., Sikorska K., Schagen van Leeuwen J.H., Schreuder H.W.R., Hermans R.H.M., De Hingh I.H., Van Der Velden J., Arts H.J., Massuger L.F. (2018). Hyperthermic Intraperitoneal Chemotherapy in Ovarian Cancer. N. Engl. J. Med..

[9] Lim M.C. (2025). CHIPOR, HORSE, and beyond: Unraveling the role of hyperthermic intraperitoneal chemotherapy (HIPEC) in ovarian cancer. J. Gynecol. Oncol..

[10] Fagotti A., Costantini B., Fanfani F., Giannarelli D., De Iaco P., Chiantera V., Mandato V., Giorda G., Aletti G., Greggi S. (2025). Hyperthermic Intraperitoneal Chemotherapy in Platinum-Sensitive Recurrent Ovarian Cancer: A Randomized Trial on Survival Evaluation (HORSE; MITO-18). J. Clin. Oncol..

[11] Zivanovic O., Chi D.S., Zhou Q., Iasonos A., Konner J.A., Makker V., Grisham R.N., Brown A.K., Nerenstone S., Diaz J.P. (2021). Secondary Cytoreduction and Carboplatin Hyperthermic Intraperitoneal Chemotherapy for Platinum-Sensitive Recurrent Ovarian Cancer: An MSK Team Ovary Phase II Study. J. Clin. Oncol..

[12] Classe J.-M., Meeus P., Hudry D., Wernert R., Quenet F., Marchal F., Houvenaeghel G., Bats A.-S., Lecuru F., Ferron G. (2024). Hyperthermic intraperitoneal chemotherapy for recurrent ovarian cancer (CHIPOR): A randomised, open-label, phase 3 trial. Lancet Oncol..

[13] Della Corte L., Conte C., Palumbo M., Guerra S., Colacurci D., Riemma G., De Franciscis P., Giampaolino P., Fagotti A., Bifulco G. (2023). Hyperthermic Intraperitoneal Chemotherapy (HIPEC): New Approaches and Controversies on the Treatment of Advanced Epithelial Ovarian Cancer-Systematic Review and Meta-Analysis. J. Clin. Med..

[14] Koole S., van Stein R., Sikorska K., Barton D., Perrin L., Brennan D., Zivanovic O., Mosgaard B.J., Fagotti A., Colombo P.-E. (2020). Primary cytoreductive surgery with or without hyperthermic intraperitoneal chemotherapy (HIPEC) for FIGO stage III epithelial ovarian cancer: OVHIPEC-2, a phase III randomized clinical trial. Int. J. Gynecol. Cancer.

[15] https://www.omnicoreagency.com/youtube-statistics/.

[16] Kumar N., Pandey A., Venkatraman A., Garg N. (2014). Are video sharing Web sites a useful source of information on hypertension?. J. Am. Soc. Hypertens..

[17] Morra S., Ruvolo C.C., Napolitano L., La Rocca R., Celentano G., Califano G., Creta M., Capece M., Turco C., Cilio S. (2022). YouTube^TM^ as a source of information on bladder pain syndrome: A contemporary analysis. Neurourol. Urodyn..

[18] Di Bello F., Ruvolo C.C., Cilio S., La Rocca R., Capece M., Creta M., Celentano G., Califano G., Morra S., Iacovazzo C. (2022). Testicular cancer and YouTube: What do you expect from a social media platform?. Int. J. Urol..

[19] Ruvolo C.C., Califano G., Tuccillo A., Tolentino S., Cancelliere E., Di Bello F., Celentano G., Creta M., Longo N., Morra S. (2022). “YouTube™ as a source of information on placenta accreta: A quality analysis”. Eur. J. Obstet. Gynecol. Reprod. Biol..

[20] Cilio S., Ruvolo C.C., Turco C., Creta M., Capece M., La Rocca R., Celentano G., Califano G., Morra S., Melchionna A. (2023). Analysis of quality information provided by “Dr. YouTubeTM” on Phimosis. Int. J. Impot. Res..

[21] Shoemaker S.J., Wolf M.S., Brach C. (2014). Development of the Patient Education Materials Assessment Tool (PEMAT): A new measure of understandability and actionability for print and audiovisual patient information. Patient Educ. Couns..

[22] Patient Education Materials Assessment Tool for Audiovisual Content (PEMAT A/V). https://www.ahrq.gov/health-literacy/patient-education/pemat2.html.

[23] Charnock D., Shepperd S., Needham G., Gann R. (1999). DISCERN: An instrument for judging the quality of written consumer health information on treatment choices. J. Epidemiol. Community Health.

[24] The DISCERN questionnaire. https://www.ndph.ox.ac.uk/research/research-groups/applied-health-research-unit-ahru/discern.

[25] Lim M.C., Chang S.-J., Park B., Yoo H.J., Yoo C.W., Nam B.H., Park S.-Y., Collaborators H.F.O.C., Seo S.-S., Kang S. (2022). Survival After Hyperthermic Intraperitoneal Chemotherapy and Primary or Interval Cytoreductive Surgery in Ovarian Cancer. JAMA Surg..

[26] Moss E., Taylor A., Andreou A., Ang C., Arora R., Attygalle A., Banerjee S., Bowen R., Buckley L., Burbos N. (2024). British Gynaecological Cancer Society (BGCS) ovarian, tubal and primary peritoneal cancer guidelines: Recommendations for practice update 2024. Eur. J. Obstet. Gynecol. Reprod. Biol..

[27] Kobayashi Y., Shimada M., Tamate M., Cho H.W., Zhu J., Chou H.-H., Kajiyama H., Okamoto A., Aoki D., Kang S. (2024). Current treatment strategies for ovarian cancer in the East Asian Gynecologic Oncology Trial Group (EAGOT). J. Gynecol. Oncol..

[28] Tokunaga H., Mikami M., Nagase S., Kobayashi Y., Tabata T., Kaneuchi M., Satoh T., Hirashima Y., Matsumura N., Yokoyama Y. (2021). The 2020 Japan Society of Gynecologic Oncology guidelines for the treatment of ovarian cancer, fallopian tube cancer, and primary peritoneal cancer. J. Gynecol. Oncol..

[29] Kim J.H., Chun S.-Y., Lee D.-E., Woo Y.H., Chang S.-J., Park S.-Y., Chang Y.J., Lim M.C. (2023). Cost-effectiveness of hyperthermic intraperitoneal chemotherapy following interval cytoreductive surgery for stage III-IV ovarian cancer from a randomized controlled phase III trial in Korea (KOV-HIPEC-01). Gynecol. Oncol..

[30] Ugurlu G.A., Ugurlu B.N. (2025). Evaluating the quality and reliability of youtube videos on tympanostomy tubes: A comprehensive analysis for patients and parents. BMC Public Health.

[31] Bulut M., Reyhan A.H. (2025). A Quality Assessment of YouTube Videos on Chalazia: Implications for Patient Education and Healthcare Professional Involvement. Cureus.

[32] Melchionna A., Ruvolo C.C., Capece M., La Rocca R., Celentano G., Califano G., Creta M., Napolitano L., Morra S., Cilio S. (2023). Testicular pain and youtube™: Are uploaded videos a reliable source to get information?. Int. J. Impot. Res..

[33] Pezone G., Ruvolo C.C., Cilio S., Fraia A., Di Mauro E., Califano G., Passaro F., Creta M., Capece M., La Rocca R. (2024). The spreading information of YouTube videos on Phosphodiesterase 5 inhibitors: A worrisome picture from one of the most consulted internet source. Int. J. Impot. Res..

[34] Morra S., Napolitano L., Ruvolo C.C., Celentano G., La Rocca R., Capece M., Creta M., Passaro F., Di Bello F., Cirillo L. (2022). Could YouTubeTM encourage men on prostate checks? A contemporary analysis. Arch. Ital. Urol. Androl..

[35] Xu T., Zhao H. (2025). Analysis of the quality of vulvar cancer-related videos on YouTube. Transl. Cancer Res..

[36] Meci A., Bollig C., Tseng C.C., Goyal N. (2025). Evaluating YouTube Videos for Resident Education in Free Flap Surgery. Laryngoscope Investig. Otolaryngol..

[37] Marra A., de Siena A.U., Iacovazzo C., Vargas M., Cesarano N., Ruvolo C.C., Celentano G., Buonanno P. (2025). Impact of YouTube^®^ videos on knowledge on tracheal intubation for anesthesiologist trainees: A prospective observational study. J. Anesthesia Analg. Crit. Care.

[38] El-Mahrouk M., Jaradat D., Eichler T., Sucher R., Margreiter C., Lederer A., Karitnig R., Geisler A., Jahn N., Hau H.M. (2025). “YouTube” for Surgical Training and Education in Donor Nephrectomy: Friend or Foe?. J. Med. Educ. Curric. Dev..

[39] Newton L., Monkman H., Fullerton C. (2025). Exploring Older Adult Cancer Survivors’ Digital Information Needs: Qualitative Pilot Study. JMIR Cancer.

[40] Garzón L.Q., Koranyi S., Engelmann D., Philipp R., Scheffold K., Schulz-Kindermann F., Härter M., Mehnert A. (2018). Perceived doctor-patient relationship and its association with demoralization in patients with advanced cancer. Psycho Oncol..

[41] Vizzielli G., Giudice M.T., Nardelli F., Costantini B., Salutari V., Inzani F.S., Zannoni G.F., Chiantera V., Di Giorgio A., Pacelli F. (2024). Pressurized IntraPeritoneal Aerosol Chemotherapy (PIPAC) Applied to Platinum-Resistant Recurrence of Ovarian Tumor: A Single-Institution Experience (ID: PARROT Trial). Ann. Surg. Oncol..

