# Supplementary material for: Is YouTube™ a Reliable Source of Information for the Current Use of HIPEC in the Treatment of Ovarian Cancer?
Source: Cancers (Basel). 2025 Oct 2;17(19):3222. doi: 10.3390/cancers17193222 (PMC12523930; doi:10.3390/cancers17193222)
Supplement: Supplementary file 1 [file cancers-17-03222-s001.zip › cancers-3863965-supplementary.pdf]

Table S1: Subanalysis according to Gender narrating voice of the video.

Characteristic	Both<br>N = 10 [1]	F<br>N = 19 [1]	M<br>N = 39 [1]	NO VOICE<br>N = 3 [1]	*p*-Value [2]
**Author group**					0.223
Healthcare professionals	8 (80%)	14 (74%)	36 (92%)	3 (100%)	
General Public	2 (20%)	5 (26%)	3 (7.7%)	0 (0%)	
**Target audience**					0.099
Healthcare workers	7 (70%)	6 (32%)	18 (46%)	0 (0%)	
General Public	3 (30%)	13 (68%)	21 (54%)	3 (100%)	
**PEMAT_UNDERSTANDABILITY (%)**	54 (31, 77)	54 (31, 77)	54 (31, 62)	69 (31, 77)	0.918
**PEMAT_ACTIONABILITY (%)**	75 (31.25, 68.25)	25 (12.5, 50)	25 (25, 75)	75 (37.5,75)	0.100
**DISCERN_Section 1 (n)**	33 (27, 33)	25 (16, 31)	27 (20, 33)	19 (16, 25)	0.061
**DISCERN_Section 2 (n)**	23 (18, 32)	17 (10, 20)	20 (16, 24)	17 (13, 23)	0.020
**DISCERN_total score (n)**	58 (49, 70)	46 (27, 59)	51 (35, 60)	39 (31, 51)	0.020
**MISINFORMATION_Score (n)**	4 (2.25, 4.75)	2 (0, 2.5)	2 (1,3)	2 (1,2)	0.106
**Q1.** In which clinical scenarios should HIPEC be considered?	8 (80%)	12 (63%)	28 (72%)	2 (67%)	0.810
**Q2.** What are the potential complications associated with the HIPEC procedure?	6 (60%)	3 (16%)	13 (33%)	1 (33%)	0.121
**Q3** Should HIPEC be performed in high-volume centers with specialized expertise?	8 (80%)	6 (32%)	14 (36%)	0 (0%)	0.024
**Q4.** Is the impact of HIPEC on survival outcomes clarified?	9 (90%)	9 (47%)	22 (56%)	1 (33%)	0.123
**Q5.** Should HIPEC be offered only within the context of a randomized controlled trial (RCT)?	3 (30%)	5 (26%)	9 (23%)	0 (0%)	0.752
**Global Quality Score**					0.013
1	0 (0%)	3 (16%)	4 (10%)	0 (0%)	
2	0 (0%)	6 (32%)	6 (15%)	1 (33%)	
3	3 (30%)	5 (26%)	11 (28%)	2 (67%)	
4	4 (40%)	5 (26%)	16 (41%)	0 (0%)	
5	3 (30%)	0 (0%)	2 (5.1%)	0 (0%)	

[1] Median (IQR); n (%)
[2] Fisher's exact test; Wilcoxon rank sum test; Welch Two Sample t-test; Pearson's Chi-squared test
